# Retrograde Optogenetics Reveals Functional Convergence within a Corticotectal Pathway of Non-Human Primates

**DOI:** 10.1101/2025.08.03.667949

**Published:** 2025-08-23

**Authors:** Xuefei Yu, Atul Gopal, Ken-ichi Inoue, Martin Bohlen, Genevieve Kuczewski, Marc A. Sommer, Hendrikje Nienborg, Masahiko Takada, Okihide Hikosaka

**Affiliations:** 1 Laboratory of Sensorimotor Research, National Eye Institute, National Institutes of Health, Bethesda, Maryland 20892, United States of America; 2 Center for the Evolutionary Origins of Human Behavior, Kyoto University, Inuyama, Aichi 484-8506, Japan; 3 Laboratory of Integrative Anatomy, Graduate School of Medical Sciences and Medical School, Nagoya City University, Nagoya, Aichi 467-8601, Japan; 4 Department of Biomedical Engineering, Duke University, Durham, North Carolina, 27708, United States of America

## Abstract

Understanding how cerebral cortex communicates with subcortical areas to drive behavior remains a central question in system neuroscience. One key unresolved issue is whether prefrontal cortical outputs to motor-related subcortical regions carry predominantly motor commands or mixed sensory - motor signals. Retrograde optogenetics offers a powerful way to interrogate such projection-defined circuits, but its use in non-human primates has been limited. Here, we applied retrograde optogenetics in awake macaques to directly test the functional organization of the corticotectal projection from the frontal eye field (FEF) to the superior colliculus (SC). We asked whether FEF output to SC is motor-dominant or reflects sensory–motor convergence. Optical activation of this pathway evoked robust, contralateral saccades and selectively modulated reaction times, demonstrating its causal role in saccade generation. Optogenetically tagging FEF neurons projecting to SC revealed a heterogeneous population of visual, visuomotor, and motor neurons. This diverse output converged predominantly onto motor-related neurons in the SC. These findings support a visuomotor convergence model and resolve long-standing questions over the composition of FEF outputs. Additionally, our results establish retrograde optogenetics as a tool for dissecting projection-defined circuits in primates and for precisely probing the neural pathways that link perception to action.

## Introduction

What information does the cerebral cortex transmit to subcortical structures to guide actions? This fundamental question is central to understanding how distributed and complex brain networks convert perception and cognition into action. In non-human primates, the prefrontal cortex is often regarded as a core region where diverse sensory and contextual information is flexibly integrated to guide decision and actions^1^. However, whether its output to subcortical action-oriented areas primarily conveys the *results* of this integration, i.e. motor-dominant signals, or instead an *intermediate stage*, i.e. sensory-motor signals, remains debated.

One example from everyday life is visual inspection of a scene and the decision where to look next. This process is thought to rely largely on output from the frontal eye field (FEF), a prefrontal region critical in cognitive control^2^, to the superior colliculus (SC), a brainstem structure essential for generation of eye movement^3^. Early studies portrayed this projection as carrying predominantly motor commands^4^, whereas subsequent research indicated a richer signal composition, encompassing visual, visuomotor, and motor signals^5,6^. The functional makeup of the FEF–SC projection therefore remains unclear. Moreover, FEF-SC signals have never been directly characterized in parallel with causal tests of their role on behavior. Resolving the nature of FEF-SC signals, how they are refined for motor control subcortically, and their importance for behavior would provide a well-defined, valuable test case for understanding perception-action circuits in the primate brain.

What has been lacking to accomplish such a cohesive study is a single technique capable of accessing SC-projecting neurons in the FEF, recording them, and stimulating them selectively. Recent advances in viral engineering^7–10^ have positioned retrograde optogenetics as a powerful solution to address this challenge by enabling projection-specific access and manipulation. This technique employs viral vectors transported retrogradely from axon terminals to somata, thereby enabling selective stimulation of projection-defined neurons via light^11^. Although widely adopted in rodents, optogenetics in non-human primates has been challenging and the specific application of retrograde optogenetics for projection-selective recording and behavioral manipulation has been largely unexplored^11–14^.

In this study, we applied retrograde optogenetics to selectively label FEF neurons projecting to the SC in macaques, aiming to resolve the longstanding questions of FEF output signals to the SC and their influence on saccades. To this end, we selectively optically stimulated the FEF-SC pathway and determined its influence on saccade generation, and identified the nature of the signal components it conveys as well as the functional subtype of SC neurons receiving these inputs. We showed that retrograde optogenetics enabled precise access to SC-projecting FEF neurons, whose selective activation profoundly modulated saccades. Recordings from the optically modulated neurons revealed that SC-projecting FEF neurons convey diverse visual, visuomotor, and motor signals, which converge onto motor-related SC neurons. Our findings support a model of visuomotor convergence in the FEF–SC pathway and establish a generalizable framework for applying retrograde optogenetics to dissect the causal roles of defined long-range projections in the primate brain.

## Results

### Pronounced and selective behavioral modulation by retrograde optogenetics

**Figure 1.**
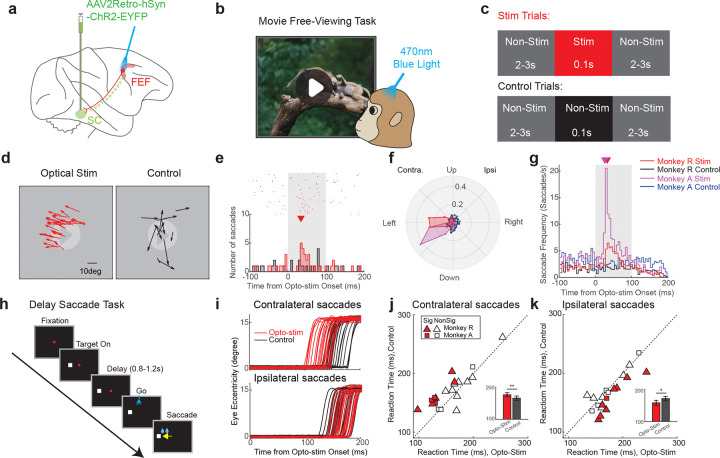
Robust saccade modulation induced by optical stimulation of the FEF-SC pathway. **a** Schematic illustration of the experimental design. The retrograde viral vector was injected into the SC, from where it was transported retrogradely to the somata of neurons in the FEF, thereby tagging the projection neurons to the SC. Optical stimulation was applied in the FEF. **b** Illustration of the movie free-viewing task. Macaques viewed movies and were rewarded for keeping their gaze within the display. **c** Optical stimulation protocol during the movie free-viewing task. Each free-viewing session consisted of two randomly interleaved trial types: stimulation trials, which included a 0.1s optical pulse from a blue laser (left red panel), and control trials, which did not include stimulation (right black panel). Each optical stimulation was embedded within two non-stimulated periods lasting 2–3s. **d** An example session showing saccades that occurred in stimulation trials (left) and control (right) trials during optical stimulation in the FEF. **e** Example peri-stimulus time histogram (PSTH) of saccades in the stimulation (red) and control trials (black) during optical stimulation in the FEF (N = 40 trials per condition). Upper raster shows time of saccade onsets in individual trials (both spontaneous and putatively stimulation-evoked). Lower histogram shows distributions of those onset times binned at a 5ms intervals. The triangle indicates the time point of peak saccade occurrence (Peak time: 35ms). **f** Polar histograms of saccade directions in stimulation and control trials for two monkeys during optical stimulation in the FEF. The histogram is binned in 22.5° intervals. The radial (ρ) axis represents the proportion of saccades within each angular bin, normalized to the total number of saccades recorded during the 100ms interval under each condition for each monkey. **g** Population PSTH of saccades in stimulation and control trials for two monkeys during optical stimulation in the FEF. To generate the PSTH, saccades were first counted and averaged across trials in 5ms bins from −100ms to 200ms relative to stimulation onset for each session. The average counts were then divided by the 5ms bin duration to yield the saccade frequency. The triangle indicates the time point of peak saccade occurrence for monkey R (red: 32.5ms) and monkey A. (magenta: 27.5ms) **h** Schematic of the delayed saccade paradigm. In each trial, a target appeared in either the left or right visual field, followed by a delay period of 0.8–1.2s. After the delay indicated by disappearance of the fixation point, the animal was required to make a saccadic eye movement to the target location. **i** An example session showing eye movement traces for contralateral (top) and ipsilateral saccades (bottom) during optical stimulation (red) and control (black) trials. Contralateral: Reaction Time (RT) for Optical stim: 122.9 ± 8.1ms [Mean ± S.E.M] N = 20 trials; RT for Control: 154.4 ± 3.3ms, N = 20 trials; p = 7.4516e-04, two sample two-tailed t-test. Ipsilateral: RT for Optical stim: 170.4 ± 5.7ms, N = 20 trials. RT for Control: 157.6 ± 5.7ms, N = 20 trials; p = 0.06, two sample two-tailed t-test. **j** Scatter plot of reaction times (RTs) for contralateral saccades in the optogenetic stimulation versus the control condition. N=20 sessions. The inset displays a bar plot for the mean RT for each condition. Mean RT during Optical stim: 158.23 ± 7.81ms, [Mean ± S.E.M]; Mean RT during Control: 171.44 ± 6.89ms; p = 0.0068, two-tailed paired t-test. Filled Symbols: Sessions with a significant difference in RT between control and optogenetic stimulation (p < 0.05, one-tailed two sample t-test); Open Symbol: No significant difference (p > 0.05). Triangle: Monkey R; Square: Monkey A. **k** Scatter plot of reaction times for ipsilateral saccades in the optogenetic stimulation versus the control condition. N = 20 sessions. The inset displays a bar plot for the mean RT for each condition. Mean RT during Optical stim: 176.43 ± 6.54ms; Mean RT during Control: 166.04 ± 6.69ms; p = 0.0084, two-tailed paired t-test.

**Figure 2.**
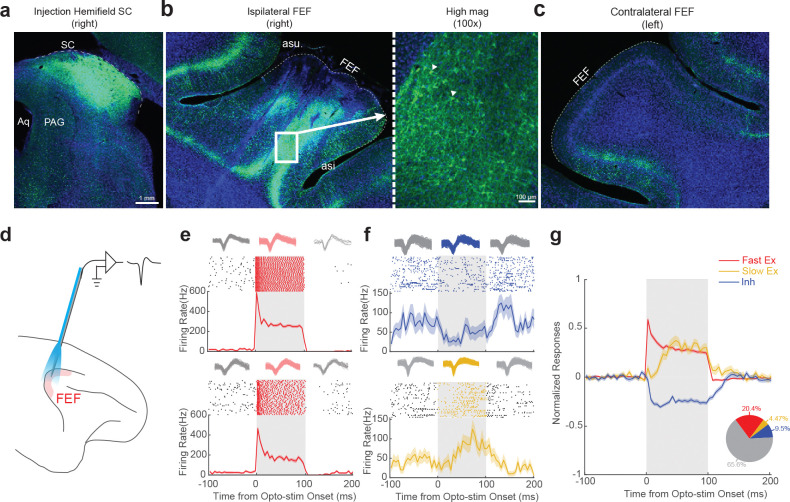
AAV2retro efficiently labeled neurons within the Frontal Eye Field (FEF)-Superior Colliculus (SC) pathway. **a** Immunofluorescence showing ChR2-EYFP expression in the injected hemifield of SC. **b** Immunofluorescence showing retrograde labeling of ipsilateral FEF neurons by ChR2-EYFP. The right panel is the magnified window(100X) showing the labeled somata. The triangles indicate two examples. **c** Lack of immunofluorescence in the contralateral FEF. Weak, expected labeling was observed in the adjacent area, ventrolateral prefrontal cortex (area 45), which is known to project to the SC^36^. **d** A schematic illustrating the experiment involving optical stimulation and simultaneous electrical recording in the FEF. **e** Two examples of putative direct activation by optical stimulation, in which neurons showed rapid (Latency: 0ms) and robust activation. The top panels show the spike waveforms for each unit: red traces represent waveforms recorded during optical stimulation, while grey traces represent those recorded outside the stimulation period. The action potential waveforms remained consistent during optical stimulation, indicating stable unit identity. **f** Two examples of putative indirect activation by optical stimulation. The top example shows inhibition (top, Latency 15ms) and the bottom one shows delayed activation (Latency: 32ms). The top panels show the waveforms for each unit. Similar to panel **e,** the waveforms remained consistent during optical stimulation. **g** Population average across three groups of neurons in the FEF based on optical stimulation effects and modulation latency. The raw peristimulus histogram (PSTH) for each neuron was normalized by dividing by its peak firing rate. Subsequently, the average baseline activity (−100ms to 0ms relative to laser onset) was subtracted, resulting in a normalized firing rate ranging approximately from −1 to 1. The pie chart in the bottom-right corner shows the proportion of FEF neurons in each group. Fast Excitation—Fast Ex (Red): N = 146; Slow Excitation—Slow Ex (Orange): N = 32; Inhibition—Inh (Blue): N = 68; Not Effective—No effect (Grey): N = 470.

We injected AAV2retro-hSyn-ChR2-EYFP unilaterally into the SC of two monkeys (Fig. 1a). To confirm gene expression in the FEF-SC projection, we successfully manipulated neural activity and saccadic behavior using fiber optic illumination of the SC (Supplementary figs. 1A–H) and showed post-mortem expression of the ChR2-EYFP complex via histological analysis of the SC (Fig. 2a) and FEF. Expression was profuse in the ipsilateral FEF (Fig. 2b) but negligible in the contralateral FEF (Fig. 2c).

Our first main experimental goal was to optically stimulate the FEF to establish that selective activation of retrogradely labeled FEF-to-SC neurons could evoke saccades. To test this, the monkeys freely viewed a movie (Fig. 1b), while 0.1s optical stimulation pulses (470nm, 50mW/mm^2^) were delivered intermittently to the FEF (Fig. 1b and 1c). Saccades during the 0.1 sec stimulation period were compared between randomly interleaved “Stim Trials” (Fig. 1c, top center) and non-stimulated “Control Trials” (Fig. 1c, bottom center) for each session.

Two key observations emerged. First, across 110 sessions, saccade frequency significantly increased during the optical stimulation period (4.24 saccades/s, N = 2443 saccades) relative to the control period (2.41 saccades/s, N = 1408 saccades, p = 4.28e-11, two-tailed paired t-test). Second, evoked saccades were consistently directed toward the left visual field (Fig. 1d shows saccades for an example session). Since optical stimulation was applied to the right FEF, these leftward saccades were contralateral to the site of stimulation (see also Supplementary fig. 2a), resembling the effects of electrical stimulation at the same sites (Supplementary fig 2b–d). The onset latency of these example optically evoked saccades from FEF was 35ms post-stimulation (Fig. 1e), about 12ms longer than the latency observed from optical stimulation in SC in the same monkey (22.5ms, Supplementary figs. 1f and 1h. Saccadic vectors evoked from optical stimulation in FEF and the SC were generally well aligned (Fig. 1f compared with Supplementary fig. 1g). The FEF optical stimulation results were highly reproducible across sessions and animals (Fig. 1f, Supplementary fig. 2a), with a consistent latency peak ranging from 27.5 ms to 32.5ms following laser onset (Fig. 1g), compared with 22.5ms latency from SC (Supplementary fig. 1h). This 5 – 10ms latency difference is consistent with axonal conduction and synaptic delay through the FEF-SC pathway^15,16^. Optical stimulation of the FEF in fixation tasks also induced consistent contralateral saccades, though with smaller amplitudes and reduced frequency (Supplementary figs. 2e–j). Importantly, no such effects were observed prior to the viral vector injection (Supplementary fig. 2k) or during red light stimulation, ineffective to activate ChR2 (Supplementary fig. 2l), confirming the specificity of the ChR2-mediated effects.

Next, we investigated whether subthreshold optical stimulation in the FEF could modulate the planning of saccades without directly evoking them, as found in electrical stimulation studies of the FEF and SC^17,18^. To do this, we applied low-intensity optical stimulation (< 20mW/mm^2^) during a delayed saccade task (Fig. 1h) targeting the final phase of saccade preparation (200ms after fixation offset). For contralateral saccades, reaction times were significantly faster with stimulation (Opto: 158.23 ± 7.81ms, [Mean ± S.E.M], N = 20 sessions. Control: 171.44 ± 6.89ms; N = 20 sessions. p = 0.0068, two-tailed paired t-test, Fig. 1i and 1j). The facilitation effect was strongest when the target location was aligned with the direction of the evoked saccade vector (Supplementary fig. 2m). By contrast, ipsilateral saccades were delayed (Opto: 176.43 ± 6.54ms; Control: 166.04 ± 6.69ms, N = 20 sessions; p = 0.0084, two-tailed paired t-test; Fig 1i and 1k). This effect was most pronounced when the target was positioned opposite to the sites’ preferred direction (Supplementary fig. 2m). During this manipulation, optical stimulation did not evoke any additional saccades, confirming that the stimulation remained subthreshold and acted specifically to modulate the reaction time. Taken together, these findings demonstrated the efficacy of our approach, in that retrograde optogenetic manipulation of the FEF-SC pathway can both trigger saccades when suprathreshold (Figs. 1d–g) and modulate the saccade timing when subthreshold depending on the stimulation strength (Figs. 1h–k).

### Identification of FEF neurons that project to the SC

We then sought to identify and characterize the FEF neurons projecting directly to the SC using pathway-selective targeting with opto-tagging to determine whether the transmitted signals span the full sensorimotor continuum (visual to visuomotor to motor) or are preferentially motor-related. To accomplish this, we recorded neuronal activity in the FEF using a customized optrode (Fig. 2d), permitting simultaneous optical stimulation and electrophysiological recording^19^.

Across recording sessions, a substantial proportion of FEF neurons (34.4%, 246/716) showed robust modulation in response to optical stimulation (examples in Fig. 2e and 2f), most commonly with increased firing rates. A subset of neurons exhibited rapid and pronounced activation, with response latencies of less than 10ms following laser onset (Fig. 2e and 2g; Supplementary fig. 3a). These short latency responses—observed in 20.4% of the recorded units (146/716)—were presumed to reflect direct activation of SC-projecting FEF neurons expressing ChR2 (Note that the results were robust by using a shorter-latency cutoff, Supplementary fig. 3g left). In addition to direct activation, we also observed neurons exhibiting either inhibition (Fig 2f, upper panel) or delayed activation (Fig 2f, lower panel), consistent with indirect modulation via presumed local circuit interactions^20^ or reciprocal connections with other brain areas, e.g. the mediodorsal thalamus^21^. Beyond the different latencies, the presumed indirectly and directly modulated units also showed different strengths of optical response modulation (Supplementary fig 3b, Modulation strength: Fast Ex, 65.54 ± 7.14, N = 146; Slow Ex, 16.32 ± 3.03, N = 32; Inh, 12.44 ± 0.87, N = 68; p = 7.84e-36, Kruskal-Wallis Test). None of these effects were observed prior to the viral vector injection (Supplementary figs. 3c, 3e) or during red-light stimulation which is ineffective for ChR2 activation (Supplementary figs. 3d, 3f), ruling out nonspecific thermal effects. Subsequent analyses were focused on this putatively directly activated SC-projecting FEF neuronal population (the N = 146 “Fast Ex” FEF neurons, Fig. 2g), to investigate their functional properties in greater detail.

### Signal content of FEF neurons that project to the SC

**Figure 3.**
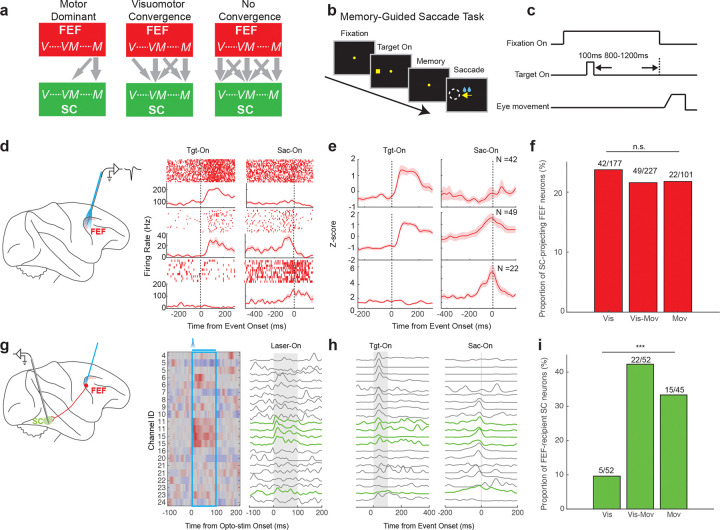
Combining optogenetic identification and behavior reveals the functional organization of the FEF-SC pathway for saccade generation. **a** Schematic illustrating possible models of information flow from the FEF to the SC. Both the FEF and SC contain visual (V), visuomotor (VM) and motor (M) related signals, but the nature of information transmitted from FEF and its target within SC remains controversial. Motor dominant model (left): FEF transmits primarily motor signals that target visuo-movement and movement neurons. Visuomotor convergence model (middle): FEF transmits a full spectrum of signals from visual to motor, and it converges onto movement related neurons in the SC. No convergence model (right): the output from FEF is also a full-spectrum, but it broadly targets all signal types in the SC. Data presented in panels D-I support the Visuomotor convergence model. **b** A schematic showing the behavioral paradigm for the memory saccade task. A target was briefly presented for 100ms, and then disappeared. After a variable delay of 800–1200ms, the fixation spot disappeared, instructing the monkey to make a saccade to the remembered target location. **c** Time course of the memory saccade task. **d** Representative responses of three example SC-projecting neurons in the FEF during the memory saccade task: a visual neuron (top), a visual-movement neuron (mid), and a movement neuron (bottom). **e** Population PSTH for SC-projecting FEF neurons: visual neurons (top, N = 42/177, 23.73%), visual-movement neurons (mid, N = 49/227, 21.59%), and movement neurons (bottom, N = 22/101, 21.78%). For each neuron, the raw PSTH was normalized by z-scoring, whereby the mean firing rate was subtracted from each time point and the result was divided by the standard deviation. **f** Proportion of SC-projecting FEF neurons that were fast-excited during optical stimulation, across the three functional subtypes of recorded FEF neurons: visual neurons, visual-movement neurons, and movement neurons. The proportion for each subtype was calculated as the number of SC-projecting FEF neurons identified in each subtype (numerator above each bar) divided by the total number of FEF neurons recorded for that subtype (the denominator above each bar). This proportion definition provides an unbiased estimate of the likelihood of identifying SC-projecting neurons within each subtype, minimizing potential bias from under- or oversampling of specific subtypes. n.s.: no significant difference in the proportions among the three groups, p = 0.86, tested by chi-squared test. **g** An example session of laminar recordings in the SC during optical stimulation of the FEF, shown as a heatmap (left). Modulated units were further classified as fast excitatory units based on excitatory responses with latencies below 10ms. These units are considered putative SC neurons directly activated by FEF stimulation, representing SC targets receiving direct projections from the FEF. They are highlighted in green in the line plot (right). **h** A line plot showing the firing rate of the same SC population showing in panel **g** during the memory saccade task, aligned on target onset (left) and saccade onset (right). Green thick lines indicate channels on the laminar probe with the fast-excitation (latency < 10ms) optically modulated units. **i** Proportion of FEF-recipient SC neurons across the three functional subtypes in the SC. The definition of proportion is the same as in panel F. ***, p < 0.001, chi-squared test.

The FEF-SC pathway can be conceptualized in three alternative organizational schemes, based on the functional properties of FEF output neurons and the properties of FEF-recipient SC neurons (Fig. 3a). One possibility, consistent with motor-enriched output of FEF^4^, is the *motor dominance model.* It posits that the FEF outputs are primarily motor-related signals that target visuo-movement and movement neurons in the SC (Fig. 3a, left). On the contrary, two other possibilities align with a broad spectrum of signals leaving the FEF^5,6^. The *no convergence model* proposes that the FEF transmits the full spectrum of signals, from purely visual to purely motor, broadly targeting all SC neuron types (Fig. 3a, right). The *visuomotor convergence model* is an intermediate model and proposes a full-spectrum output from the FEF, but emphasizes convergence onto movement-related neurons in the SC (Fig. 3a, middle). Discriminating among these models requires examination of both the functional properties of SC-projecting FEF neurons and their targets within the SC.

To this end, we defined three subtypes of SC-projecting FEF neurons using a memory-guided saccade task, which was designed to dissociate sensory responses from motor-related activity^5^ (Fig. 3b and 3c). “Visual neurons” responded exclusively following target onset, without activation preceding the saccade (example in Fig. 3d top; subtype average in Fig. 3e top). “Visuo-movement neurons” showed both visual responses after target onset and motor-related activity prior to saccade initiation (Fig. 3d and 3e middle). By contrast, “movement neurons” exhibited activation only before saccade onset, with no responses to the target presentation (Fig. 3d and 3e bottom). The optically labeled SC-projecting FEF neurons were distributed in approximately equal proportions across all the three functional subtypes (Fig. 3f, p = 0.86, Chi-Squared Test), indicating that a diverse range of signal types is transmitted from the FEF to the SC. This distribution remained qualitatively similar when applying a shorter-latency cutoff of < 5ms to define SC-projecting FEF neurons (Fig. 3g right). These findings are therefore inconsistent with the motor dominance model (Fig. 3a left).

### Signal content of SC neurons that receive FEF input

To further discriminate between the no convergence and visuomotor convergence models (Fig. 3A, right vs. middle), we next mapped the corresponding postsynaptic targets of the FEF projection neurons within the SC. For this purpose, we performed laminar recordings in the SC while optically stimulating the FEF (Fig. 3G). Out of 163 SC neurons recorded across 10 sessions, FEF stimulation modulated a substantial number (N = 64, 39.26%), with excitation being the dominant effect (81%, 52/64; Supplementary fig. 4a). To isolate putative monosynaptic FEF targets, we focused on SC neurons displaying rapid activation (Latency < 10ms; Supplementary fig. 4b,). These units were primarily recorded from the middle channels of the SC probe (Fig. 3g), consistent with the SC intermediate layers. To quantify their functional properties, we again used the memory-guided saccade task and applied the same classification as described above (example raw data across channels in Figure 3H). Within each class, we examined the proportion of rapidly activated units. The vast majority of activated SC neurons were either visuomotor (N = 22/52, 42.3%) or motor (N = 15/45, 33.3%) in nature, whereas visual-only neurons were rare among the FEF-recipient population (N = 5/52, 9.6%; Fig.3i, p = 9.86e-06, Chi-Squared Test). This functional bias could not be explained by sampling artifacts, because our recordings sampled comparable numbers of visual (N = 52), visuo-movement (N = 52) and movement neurons (N = 45), nor could it be attributed to the injection variability, as direct optical stimulation of the SC produced a balanced distribution of all three subtypes (Supplementary fig. 1d). Hence our results support the visuomotor convergence model: while the FEF output is functionally heterogeneous and conveys a continuous spectrum of signals, it preferentially targets visuo-movement and movement neurons in the SC (Fig. 3c middle).

## Discussion

Using retrograde optogenetics, we selectively manipulated and identified the projections from the FEF to the SC, revealing that the FEF-SC pathway transmits a continuous spectrum of sensorimotor signals ranging from visual, visuomotor and motor. This result helps reconcile previous conflicting findings derived from electrical antidromic stimulation approaches^4,6^. Examining the SC targets of these projections, we found that FEF inputs converge primarily onto motor-related neurons in the SC, consistent with earlier anterograde stimulation studies^22^. Crucially, retrograde optogenetics offers superior pathway specificity: unlike antidromic stimulation, which may nonspecifically activate nearby axons or unrelated pathways^23^, retrograde viral vectors ensure only the intended projection is labeled and manipulated^11^. This methodological precision likely explains discrepancies with prior approaches and underscores the advantages of our strategy.

Another advantage of our study lies in its integrated approach. Previous investigations about the functionality of a pathway often relied on separate experiments: antidromic stimulation was used to identify source regions^5,24,25^, orthodromic stimulation to evaluate effects to downstream targets^21,22^, and independent studies to assess behavioral modulation^26,27^. In contrast, our work simultaneously examined both the source and the target regions, along with causal manipulation of the circuit, within the same animals using consistent methodologies. Thus this pathway-selective strategy provides a clearer view of the underlying neural mechanisms supporting cognitive processes and reduces the variability associated with studies conducted across different laboratories, species, or time periods.

Our findings support the visuomotor convergence model, which posits that diverse cortical output signals converge to movement-dominant processing within the FEF-SC pathway. This functional convergence favors a view of sensorimotor processing as a continuous, multistage transformation, where activity at any stage along the pathway can advance the computation toward action^24^. Thus, the present results are also consistent with cognitive models proposing graded transitions between perception and action^28,29^. Future work will be needed to determine whether this organizational principle—convergence of diverse cortical output signals onto motor representations in subcortical structures—can be generalized across cortico-subcortical circuits in other sensorimotor systems.

Using the primate oculomotor system as a model, our study also highlights the utility of retrograde optogenetics as a powerful tool for simultaneously manipulating behavior and labeling pathway-defined neuronal populations. The selective manipulation of behavior is a critical foundation for probing higher cognitive functions^30^. While prior optogenetic studies in non-human primates have established causal links between neural activity and behaviors, these efforts have largely relied on local stimulation at injection sites^31^ or anterograde manipulation^27,32^. Our study fills a critical gap by achieving robust and selective manipulation of behavior via retrograde, pathway-selective optogenetics. This approach yields behavioral effects comparable in strength to classical methods including electrical stimulation, but with far greater specificity by targeting pathway-defined neuronal subpopulations. Recent histological studies in macaques have shown that AAV2-retro enables reliable retrograde labeling in several other cortical areas such as the anterior cingulate cortex, primary motor cortex, and ventrolateral prefrontal cortex^10,33^. These cortical long-range projections are all likely glutamatergic, but further studies could explore whether this approach is equally effective in targeting subcortical structures having GABAergic projections, such as the substantia nigra and zona incerta^18,34,35^. Together, the projection specificity, labeling precision, and behavioral efficacy of retrograde optogenetics make it a highly promising tool for dissecting circuit-level computations underlying cognitive processes in non-human primates, especially where functional heterogeneity and pathway specificity are essential for understanding the neural basis of behavior.

## Methods

### Subjects

Two adult male rhesus monkeys (*Macaca mulatta*, monkeys R and A), weighing 9–12kg, participated in the present study. All experimental protocols and procedurals were approved by the National Eye Institute Animal Care and Use Committee and complied with the United States Public Health Service Policy on the Humane Care and Use of Laboratory Animals.

### Surgery

We implanted a plastic head holder and two recording chambers for accessing the SC and FEF in the skull under general anesthesia and sterile surgical conditions for each monkey. The SC recording chamber was positioned on the midline and angled 38 degrees posterior to vertical on the sagittal plane, allowing electrode penetrations to approach the SC surface approximately orthogonally in both monkeys. The FEF recording chamber was placed over the frontal lobe and tilted laterally by 20 degrees. The head holder and recording chambers were secured with ceramic screws and embedded in dental acrylic resin, which covered the top of the skull. After confirming the position of recording chambers using MRI (4.7T, Bruker), a craniotomy was performed for each chamber during another surgery for future recordings.

### Behavioral tasks

All behavioral task events and data acquisition were conducted using custom-made C++ based software (Blip, http://www.robilis.com/blip/). The monkeys were seated in a primate chair with their heads restrained, facing a fronto-parallel screen in a sound-attenuated and electrically shielded room. Visual stimuli were rear projected onto the screen using a digital light processing projector (PJD8353s, ViewSonic). Eye position was monitored at a sampling rate of 1KHz using an infrared eye-tracking system (EyeLink 1000 Plus, SR Research). Water rewards were delivered through a computer-controlled electromagnetic solenoid valve (Parker-Hannifin). The timing of all task-related visual events was recorded relative to the timing of the evoked signal using a photodiode positioned at the corner of the screen.

### Behavior tasks for stimulation

We trained monkeys to perform four different tasks for evaluating the effect of optical or electrical stimulation.

#### Movie free viewing task

The subjects freely watched randomly selected natural history movies (15–25 minutes long) continuously with occasional random water rewards to keep them engaged. Unbeknownst to the subject, the entire viewing period was artificially divided into trials 2–3 seconds long separated by inter-trial intervals of 1–2 seconds. Optical (4.5mW, equals 50mW/mm^2^, 100ms) or electrical (50uA, 100Hz, 100ms) stimulation was applied in half of the total trials, at a random time point between 500ms and 1s after the start of the trials. To ensure wakefulness and engagement, the eye movements were monitored, and the subjects were required to keep their eyes open and within the designated 40° × 40° window throughout the trial. Optical or electrical stimulation was applied only when the eyes were detected within this window. If the animals moved their eyes outside the window or closed them, the current trial was aborted and repeated later. No movement constraints were imposed during the inter-trial intervals. If a subject successfully completed a trial, a small amount of water reward was delivered at the end of the trial. A typical session consisted of 80 or 160 successful trials.

#### Fixation Task

In the fixation task, subjects were required to maintain fixation throughout each trial. At the beginning of a trial, a central fixation point (1° × 1° degree) was illuminated, and the subjects had to fixate on the point within 500ms. Between 500ms and 1000ms after fixation onset, the optical stimulation was applied for 100ms in half of the trials (4.5mW, equivalent to 50mW/mm^2^, 100ms; stimulated trials), while no stimulation was applied in the other half (control trials). Stimulated and control trials were randomly interleaved. Following the stimulation period, subjects continued fixating for an additional 500ms to 1000ms, making the total fixation duration for each trial 2s. The fixation point remained visible throughout the entire fixation period. The fixation window was ±5°. Subjects were allowed to break fixation during the stimulation period (100ms) and 200ms after the stimulated period, as well as during the corresponding 200ms period in control trials. If fixation was broken during this permissive window, subjects had 500ms to re-fixate on the fixation point and complete the remaining fixation period to successfully finish the trial. Successfully maintaining fixation for the required duration resulted in a water reward. A typical session consisted of 80 or 160 trials.

#### Gap Fixation task

The procedure for the gap fixation task was nearly identical to that of the fixation task, except that the fixation point disappeared for 500ms in the middle of the trial. During training, subjects were required to maintain fixation in each trial even when the fixation point was absent. In the stimulation experiment, optical stimulation was applied 200ms after the fixation disappeared, and ended 200ms before the end of the fixation-off period. Eye movements during the stimulated period were compared with those during the corresponding fixation-off period in control trials.

#### Delayed saccade task

In the delayed saccade task, subjects were required to make a saccadic eye movement to a visual target that appeared in the periphery after a random delay. Each trial began with the presentation of a central fixation point. After subjects maintained fixation for 500ms to 1000ms, a saccade target was displayed either contralateral or ipsilateral to the stimulated hemifield. The target location was aligned with the saccade vectors elicited by stimulating during the free-viewing task. Following a delay period of 800ms to 1200ms, the fixation point was turned off, serving as the go signal instructing the subject to make a saccadic eye movement toward the target location to receive a liquid reward. Optical stimulation was applied using blue laser light for 200ms, starting with the go signal. Trials with and without stimulation were randomly interleaved. To ensure subthreshold stimulation, the laser power was reduced to less than half (< 2mW, equivalent to 20mW/mm^2^). An initial set of 200 trials was first conducted before the formal experimental session, to confirm that the stimulation did not directly trigger saccades (i.e. fewer than 1% of cases, i.e. fewer than one saccade per 100 trials). A typical recording session consisted of 160 – 320 trials in total.

### Behavior tasks for neural recordings

Once units were identified, we first manually mapped their receptive field approximately. We then fixed the target presentation eccentricity and varied the target presentation angle to determine the preferred vector using a receptive field mapping task. Following this, we employed a memory saccade task to classify the functional subtype of each unit.

#### Receptive field mapping task

The procedure of the receptive field mapping task was identical to that of the delayed saccade task except that no stimulation was applied and the target appeared at one of 8 possible locations: 0 degree (up), 45 (left - up), 90 degree (left), 135 degree (left - down), 180 degree (down), 225 degree (right - down), 270 degree (right), or 315 degree (right - up).

#### Memory-guided saccade task

The task began with the appearance of a fixation point. The subject was instructed to acquire and maintain fixation within a 5°×5° window. After holding fixation for 800ms, a target was briefly flashed for 200ms, then the fixation point remained for an additional 800ms −1200ms before disappearing. The offset of the fixation point served as the go signal, prompting the subject to saccade to the remembered target location within 500ms. To successfully trigger the reappearance of the target stimulus, the saccade had to land within 5 degrees of the target location. The subject then had to maintain fixation on the target for another 400ms to receive a reward.

### Viral vector preparation

Adeno-associated viral vectors with retrograde infection expressing the channelrhodopsin gene (AAV2-Retro hSyn-ChR2-EYFP) were produced by the helper-free triple transfection procedure and purified by affinity chromatography (GE Healthcare). Viral titer was determined by quantitative PCR using Taq-Man technology (Life Technologies). The transfer plasmid (pAAV-hSyn-ChR2-EYFP-WPRE) was constructed by replacing the CMV promoter of the pAAV-CMV-ChR2-EYFP-WPRE plasmid^27^ with the human Synapsin (hSyn) promoter.

### Viral vector injection

We first identified the SC using a combination of magnetic resonance imaging and electrophysiological properties. After the identification, AAV2retro-hSyn-ChR2-EYFP (2.5 × 10^13^ genome copies/mL) was injected into the SC of one hemisphere in both monkeys (monkey R: left SC, monkey A: right SC) using a custom-made injectrode. For each monkey, two penetrations were made at least 1.4mm apart. In each penetration, 1.0ul of the vector was injected at two different depths, separated by at least 500um. The injection process began with an initial infusion of 0.2uL at a rate of 0.4uL/min, followed by a slower infusion rate 0.02uL/min for the remaining volume. This process was controlled using a 10uL Hamilton syringe and a motorized infusion pump (Harvard Apparatus, Holliston, MA, USA). After each injection, the injectrode was left at the site for 1 hour before proceeding to the next site to ensure absorption of viral solution into the surrounding neural tissue. Recording and testing experiments were conducted 2 – 3 month after injection.

### Laser light stimulation

We used custom-made single channel or multi-channel optrodes for optical stimulation and electrophysiology recordings. The single channel optrode had an optic fiber (Doric Lenses, 200um diameter, 0.5NA) attached to a tungsten recording electrode (FHC, 0.5–2MOhm impedance, 125um diameter). The tip of the electrode typically extended 200um beyond the fiber tip. The multi-channel optrodes had the same optic fiber attached to a 24-channel laminar probe (V probe, Plexon INC,100um inter-contact- distance). The tip of the fiber was placed close to the third and fourth channels on the probe. The light source was a 473nm blue diode laser with a maximum power up to 300mW (Topica photonics, iBeam smart). Before the optrode was inserted into the brain, we measured the light intensity at the tip of the optrode using an optical power meter (Newport Corporation, Centauri). During the movie free viewing task, we typically fixed the output power to be 4.5mW (~50mW/mm^2^) in FEF and 1.5mW (~16mW/mm^2^) in SC, for significant behavior and neuronal modulation.

### Electrophysiology

Electrophysiological recordings were performed using either single channel or 24-channel V probe optrodes (Plexon). Electrodes were inserted into the brain through a stainless-steel guide tube and advanced using oil-driven micromanipulator (MO-97A, Narishige). Recording sites were targeted using a grid system that enable electrode placement at 1-mm intervals in x and y directions, orthogonal to the guide tube. Extracellular signals were amplified and band-pass-filtered (100 Hz to 8kHz) using a multichannel processor (Plexon). Spike sorting was conducted offline using Kilosort^37^ and manually curated by a human expert using Phy2 (www.github.com/cortex-lab/phy) to ensure that the sorted units exhibited physiologically plausible inter-spike interval distributions and waveform shapes consistent with action potentials. Neurons were excluded from analysis if they had low signal-to-noise ratio, a low average trial firing rate (<1spikes/s), or lacked a clear visual or motor responses. The FEF was identified in the anterior bank of the arcuate sulcus, where saccades could be evoked via low threshold electrical stimulation (Threshold < 50uA, 100Hz) and neurons showed clear visual and/or saccade related responses. The SC was identified as showing clear visual and/or motor responses and clear transition from visual neurons to visual-movement neurons and to movement units during the penetration.

### General experimental procedures

During a typical recording session, once neurons were stabilized and well-isolated, the receptive field of a randomly selected neuron was initially mapped by manually placing a square target at various screen locations to identify the position that elicited the strongest neural responses. At this stage, the receptive field locations were roughly determined by subjectively evaluating the strength of the neural responses through auditory monitoring, and those responses were confirmed as either visual or saccade related. Next, the movie free viewing task was administered to assess the optical modulation of the recorded neurons. The isolated units were then tested on a subset or all of the following tasks: fixation stimulation task, gap fixation task, delayed saccade task, receptive mapping task, and the memory saccade task, among others. Neurons were recorded across all subsequent tasks regardless of whether they exhibited optical modulation, in order to avoid sampling bias.

### Dataset

In total, we recorded 716 neurons from the FEF, comprising 562 neurons from Monkey R across 82 sessions and 154 neurons from Monkey A across 28 sessions, to evaluate the effects of optical modulation during the movie free-viewing task. Of these, 612 neurons were also recorded during the memory saccade task for functional subtype characterization. We excluded 30 neurons due to a mean firing rate of less than 1 spike/s during the task period, and 77 neurons due to the absence of significant visual or saccade-related responses. This resulted in 505 neurons being included for further functional subtype characterization. To assess optical modulation effects in the SC, we recorded 368 neurons (285 from Monkey R in 37 sessions and 83 from Monkey A in 8 sessions). Among these, 323 neurons were also recorded during the memory saccade task. We excluded 23 neurons for low firing rate (< 1 spike/s) and 59 neurons for lacking significant visual or saccade related activity, resulting in 241 SC neurons retained for functional subtype characterization. In addition, we recorded 163 extra SC neurons in 10 sessions while conducting optical stimulation in the FEF, of which 149 were also recorded during the memory saccade task and used for functional subtype characterization.

### Data analysis

#### Saccade analysis

In the free-viewing task, eye traces spanning 300ms—comprising 100ms before optical stimulation onset, the 100ms optical stimulation period or un-stimulated control period, and the 100ms after stimulation offset—were extracted for saccade analysis. Raw eye position traces were differentiated to obtain velocity and acceleration profile. Saccade detection was based on a modified version of the algorithm described previously^38^. Briefly, candidate saccades were identified when eye velocity exceeded 200 degrees/s and acceleration exceeded 1000 degree/s^2^. Saccades were then detected as the time point when velocity surpassed a threshold of 40 degrees/s. Saccade onset was defined as the time point when velocity first exceeded 40 degrees/s and offset was defined as the time point when velocity fell below this threshold. Sequential saccades were considered valid only if the inter-saccade interval was at least 20ms. For each detected saccade, direction was calculated as the vector from the saccade start to end point, and amplitude was defined as the Euclidean distance between the saccade start to the end point. To minimize noise, only saccades with amplitudes between 5 to 40 degrees were included in the analysis for this task. In the delayed saccade task, reaction time was defined as the interval between the offset of the fixation point and the onset of the saccade towards the visual target.

#### Optical modulation analysis

For optical stimulation experiments, the neural responses of FEF during the 100ms stimulation period were compared to the corresponding 100ms period in the non-stimulated (control) trials. Neurons were labeled as optically modulated if their average responses during stimulation differed significantly from those in control trials, tested via a two-sample t-test using a criterion of p<.05. Neurons showing higher activity during stimulation compared to control were classified as excitatory modulated, while those with lower activity were classified as inhibitory modulated. For significantly modulated neurons, we then measured the modulation latency. Raw spike rasters were divided into two segments: pre-stimulation and stimulation (from onset to offset). Within the stimulation segment, we compared the spike counts in each 10ms bin to the 10ms bin immediately preceding stimulation onset using a paired t-test. The 10ms window was advanced in 1ms steps for successive comparisons. If 5 consecutive windows showed significant differences, the latency was defined as the onset time of the first significant window. The latency was marked as NaN and excluded from the significantly modulated group if no significant window was detected by the end of the stimulation period. Based on the latency, the excitatory modulated population was further divided into 2 groups: (1) fast excitatory neurons with latency <10ms; (2) slow excitatory neurons with latency >=10ms.

#### Functional subtype characterization

We used spike rates from three time windows in the memory saccade task to categorize neurons in the FEF, following the criteria defined previously^39^: a baseline window (−100ms to −50ms relative to target onset), a visual response window (+60ms to +100ms after target onset), and a pre-saccadic window (−100ms from saccade onset up to saccade offset). For each neuron, a one-way Kruskal-Wallis nonparametric ANOVA was performed across trials using spike rates from the three time windows. Post hoc comparisons were then used to assess whether the visual and/or saccadic activity differed significantly from baseline. Neurons showing significant visual but not saccadic modulation were labeled visual neurons (N = 177). Neurons with significant activity in both visual and saccade windows were classified as visual-movement neurons (N = 227). Neurons with significant activity only in the saccade window were labeled movement neurons (N = 103). The characterization for SC neurons used the same approach but with a time window adjusted according to the response properties of SC neurons: baseline window: −100 to −50ms relative to target onset; visual response window: +30 to +100ms after target onset, and the pre-saccadic window: −50ms from saccade onset up to saccade offset.

### Histology

After completing all experiments, monkey R was sedated with ketamine HCl and then deeply anesthetized with an overdose of sodium pentobarbital (50 mg/kg, intravenous). Once the animal reached a fully areflexic state, the transcardial perfusion was performed, beginning with 6 liters of 0.1 M phosphate-buffered saline (PBS, pH7.2), followed by 4L of 4% paraformaldehyde in 0.1M PBS (pH7.2). After perfusion, the brain was fixed to the stereotaxic frame and cut into blocks in the coronal plane including the midbrain regions. These blocks were postfixed in 4% paraformaldehyde overnight at 4°C, then cryoprotected in a 30% sucrose solution in 0.1 m PBS at 4°C. Once fully saturated and sunk, the tissue was frozen and serially sectioned into 50um slices using a freezing stage sliding microtome (American Optical Company). The tissue sections were then stored in PBS at 4°C until processed for immunofluorescence.

Sections were divided into six evenly spaced series. One series was reserved for assessing endogenous fluorescence while the other five were subjected to immunofluorescent amplification. Detailed procedures for immunofluorescent amplification have been previously published^40^. Briefly, serial free-floating sections were rinsed in PBS, permeated with 0.5% Triton-X 100 in PBS, blocked with 1% Bovine Serum Albumin and Triton-X 100 solution, and incubated in Goat Anti-GFP (1:800, Rockland Cat#: 600–101-215) antibody solution at 4°C on a rocker for 48 hours. Next, the tissue was rinsed and incubated in a secondary antibody solution of 0.5% Triton-X 100, 1% Normal Donkey Serum, and Donkey anti-Goat Alexa Fluor 488 (1:200, ThermoFisher Cat#: A-11055) at room temperature. After three hours, the sections were rinsed in PBS then incubated in Hoechst 33342 (1:1000, ThermoFisher Cat#: 62249) for 10 minutes. Finally, the tissue was rinsed in PBS and mounted on 1% gelatinized microscope slides. Mounted tissue was then dried at room temperature overnight, then dehydrated in graded ethanols (70%, 95%, 100%) and cleared with xylene. The tissue was coverslipped using Cytoseal 60. The sections were then digitized using an Axioscan.Z1 (Carl Zeiss) at 5x using an Axiocam 506 and HXP 120V as a light source using the DAPI, Green Fluorescent, and Texas Red filters.

## Supplementary Material

Supplement 1

## Data Availability

Data and code to analyze the data have been deposited in a Github repository: https://github.com/xuefeiyu2015/RetrogradeOptogenetics.git and is publicly available as of the date of publication. Any additional information required to reanalyze the data reported in this paper is available from the lead contact upon request.
