# Supplementary material for: Retrograde Optogenetics Reveals Functional Convergence within a Corticotectal Pathway of Non-Human Primates
Source: bioRxiv. 2025 Aug 4:2025.08.03.667949. Preprint. [Version 2] doi: 10.1101/2025.08.03.667949 (PMC12324461; doi:10.1101/2025.08.03.667949)
Supplement: Supplementary file 1 [file media-1.pdf]

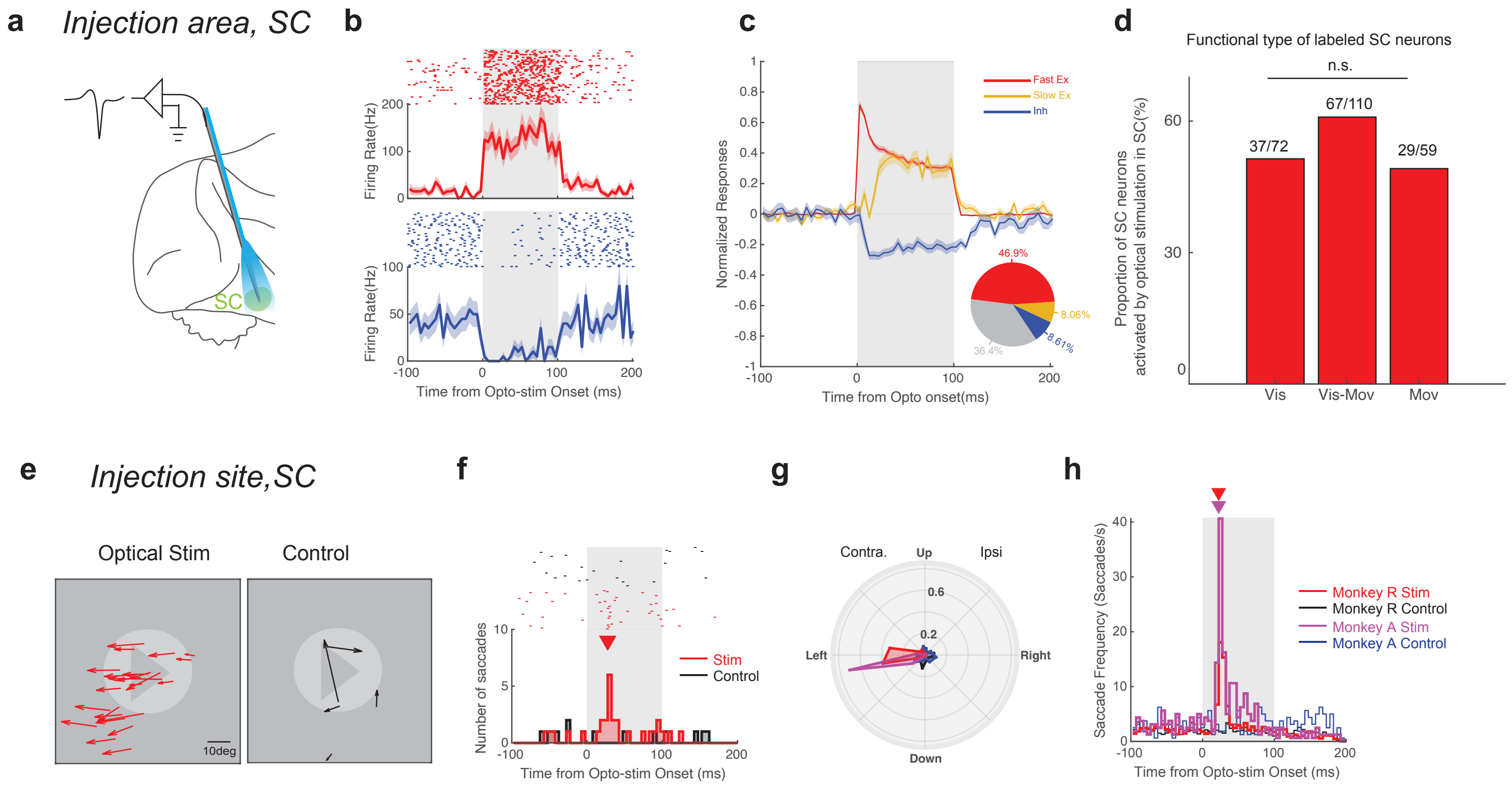

Extended Data Fig.1

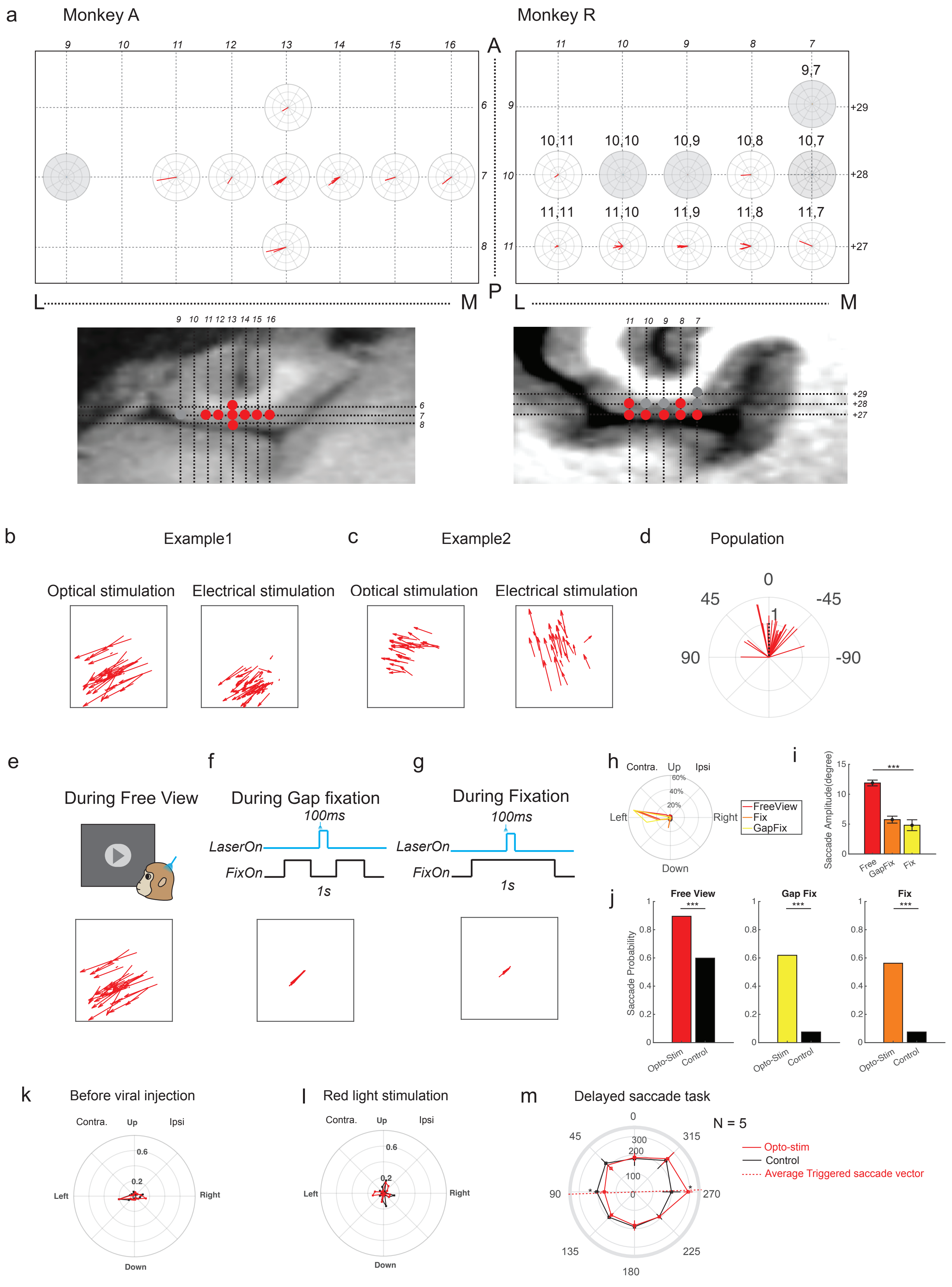

Extended Data Fig.2

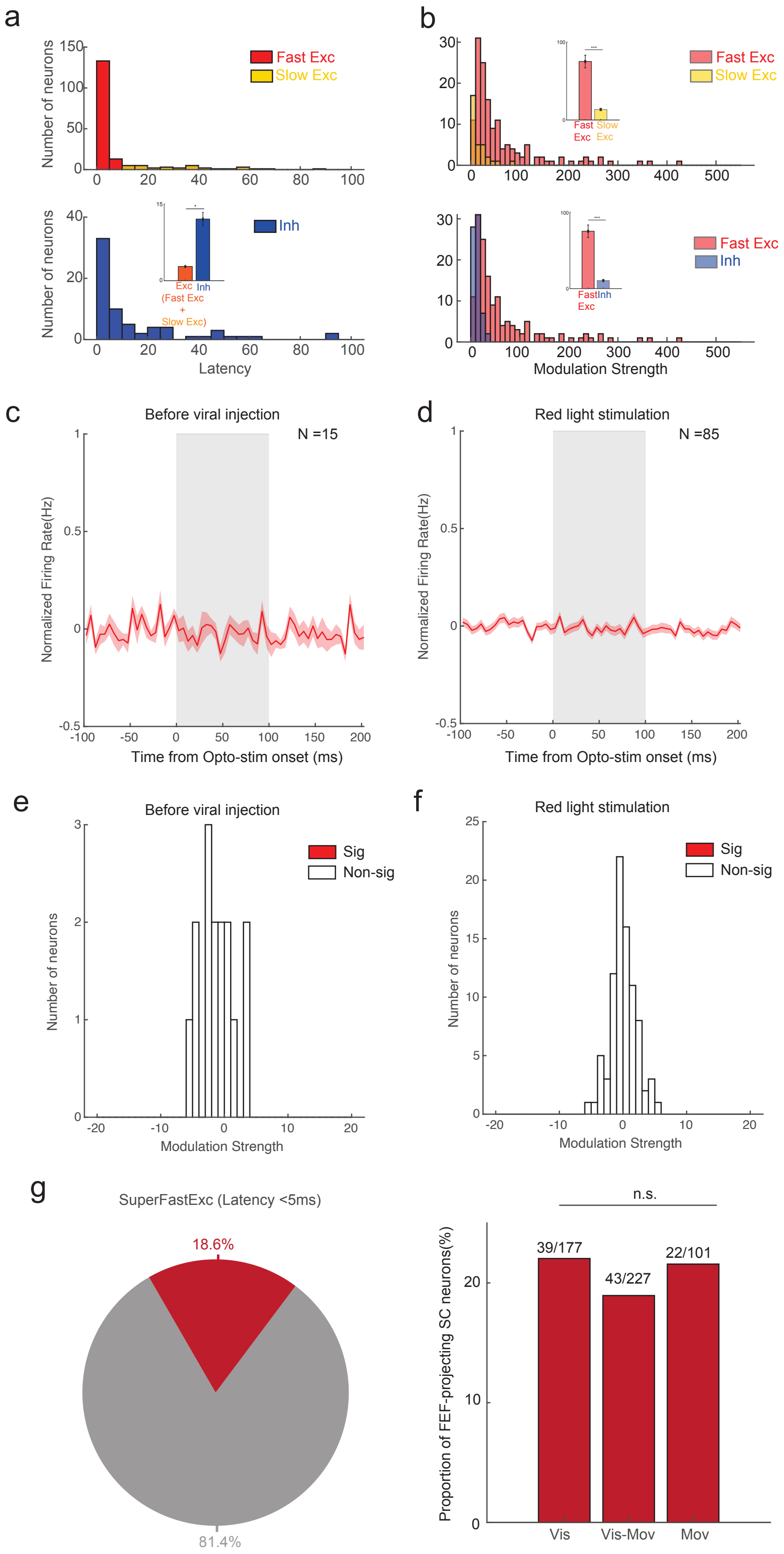

Extended Data Fig.3

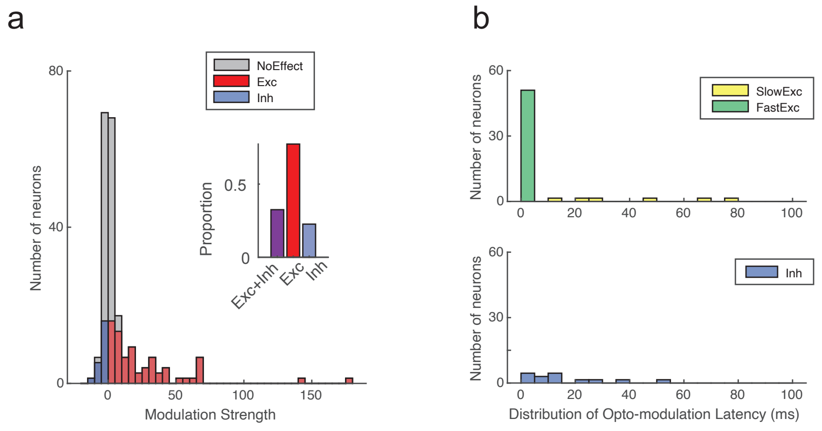

Extended Data Fig.4
